# Testing the generalizability and effectiveness of deep learning models among clinics: sperm detection as a pilot study

**DOI:** 10.1186/s12958-024-01232-8

**Published:** 2024-05-22

**Authors:** Jiaqi Wang, Yufei Jin, Aojun Jiang, Wenyuan Chen, Guanqiao Shan, Yifan Gu, Yue Ming, Jichang Li, Chunfeng Yue, Zongjie Huang, Clifford Librach, Ge Lin, Xibu Wang, Huan Zhao, Yu Sun, Zhuoran Zhang

**Affiliations:** 1 School of Science and Engineering, The Chinese University of Hong Kong, Shenzhen, China; 2 Department of Mechanical Engineering, University of Toronto, Toronto, Canada; 3 Institute of Reproductive and Stem Cell Engineering, School of Basic Medical Science, Central South University, Changsha, China; 4 Reproductive & Genetic Hospital of Citic-Xiangya, Changsha, China; 5 School of Medicine, The Chinese University of Hong Kong, Shenzhen, China; 6 Suzhou Boundless Medical Technology Ltd., Co., Suzhou, China; 7 CReATe Fertility Centre, Toronto, Canada; 8 The 3rd Affiliated Hospital of Shenzhen University, Shenzhen, China; 9 Department of Computer Science, University of Toronto, Toronto, Canada; 10 Institute of Biomedical Engineering, University of Toronto, Toronto, Canada; 11 Department of Electrical and Computer Engineering, University of Toronto, Toronto, Canada

**Keywords:** Semen analysis, Sperm detection, Generalizability, Multicenter validation, Deep learning

## Abstract

**Background:**

Deep learning has been increasingly investigated for assisting clinical in vitro fertilization (IVF). The first technical step in many tasks is to visually detect and locate sperm, oocytes, and embryos in images. For clinical deployment of such deep learning models, different clinics use different image acquisition hardware and different sample preprocessing protocols, raising the concern over whether the reported accuracy of a deep learning model by one clinic could be reproduced in another clinic. Here we aim to investigate the effect of each imaging factor on the generalizability of object detection models, using sperm analysis as a pilot example.

**Methods:**

Ablation studies were performed using state-of-the-art models for detecting human sperm to quantitatively assess how model precision (false-positive detection) and recall (missed detection) were affected by imaging magnification, imaging mode, and sample preprocessing protocols. The results led to the hypothesis that the richness of image acquisition conditions in a training dataset deterministically affects model generalizability. The hypothesis was tested by first enriching the training dataset with a wide range of imaging conditions, then validated through internal blind tests on new samples and external multi-center clinical validations.

**Results:**

Ablation experiments revealed that removing subsets of data from the training dataset significantly reduced model precision. Removing raw sample images from the training dataset caused the largest drop in model precision, whereas removing 20x images caused the largest drop in model recall. by incorporating different imaging and sample preprocessing conditions into a rich training dataset, the model achieved an intraclass correlation coefficient (ICC) of 0.97 (95% CI: 0.94-0.99) for precision, and an ICC of 0.97 (95% CI: 0.93-0.99) for recall. Multi-center clinical validation showed no significant differences in model precision or recall across different clinics and applications.

**Conclusions:**

The results validated the hypothesis that the richness of data in the training dataset is a key factor impacting model generalizability. These findings highlight the importance of diversity in a training dataset for model evaluation and suggest that future deep learning models in andrology and reproductive medicine should incorporate comprehensive feature sets for enhanced generalizability across clinics.

**Supplementary Information:**

The online version contains supplementary material available at 10.1186/s12958-024-01232-8.

## Introduction

Deep learning has been increasingly applied to facilitate diagnosis and treatment of various diseases [1, 2]. Taking infertility as an example, which affects one in six couples worldwide [3, 4], numerous deep learning models have been developed with the aim of improving clinical outcomes and optimizing the operational efficiency in in vitro fertilization (IVF) clinics [5–8]. Most of these models take images as input, for instance, to evaluate sperm motility, concentration, and morphology for selecting high-quality sperm for fertilization [9–11] or for diagnosing male infertility [12–14], to help identify and distinguish sperm and debris in testicular sperm samples [15, 16], or to examine the quality of oocytes [17]. Models have also been developed to use embryo images or time-lapse videos to grade embryos [18, 19] and to predict treatment outcomes such as implantation [20], pregnancy [21], and live birth [22–24].

Despite the potential of deep learning models for advancing clinical practice, existing studies focused on improving model accuracy [25–28] or precision [29–32] while little attempt has been made to investigate model generalizability, an essential aspect for deploying deep learning models for clinical applications. Translating a technique from technical development to clinical deployment can involve various factors that impact the generalizability of the developed technique. Regardless of applications or the types of cells to analyze, the first technical step for deep learning models is often to visually identify and locate an object (oocyte [33, 34], sperm [35–39], and embryo [20, 40–42]) in images. Different clinics, however, use different image acquisition conditions (e.g., microscope brands and models, imaging modes [43–45], magnifications [9, 33], illumination intensity, and camera resolutions [13–15, 39] etc.), as evident in Table 1. In addition, even though the images are acquired under the same conditions, sample preprocessing protocols may also be different among clinics (e.g., for sperm analysis using raw semen versus washed samples). These factors inevitably change the appearance of the images for analysis by deep learning models, thus raising concerns over whether the accuracy of a model reported in one clinic could be reproduced in another clinic.

This question is important but has not been investigated in literature. Existing studies [12–15, 35–39, 43–45], were retrospective studies where a retrospectively collected dataset was split into training, validation, and testing sub-datasets. Although such datasets may include data from multiple clinics [10, 11], model validation and testing were still performed under the same data collection conditions as the training dataset. The lack of prospective model validation and testing with new data beyond the retrospectively collected dataset challenges the generalizability of the developed model under different clinical setups. To address this question, what is needed is prospective validation and testing of model generalizability. However, existing studies mainly use accuracy or precision as the sole metric for evaluating the developed models. Reproducibility metrics such as coefficient of variation or intraclass correlation coefficient (ICC) has rarely been reported in literature.

Technically, applying a pre-trained model in different clinics may involve domain shift, that is, the data (in each clinic) used to evaluate the model is drawn from a population different from the training data. Despite the importance and implications of domain shift in image analysis and deep learning have been discussed in the literature [49–53], few studies have explored the specific factors that contribute to domain shift and its impact on model generalizability in real-world clinical settings. For instance, variations in clinical imaging settings – such as differences in microscope models, imaging modes, magnifications, and sample preparation protocol – may all lead to different data distributions between the testing dataset and training dataset. However, it remains unclear how each specific factor/variation affects model generalizability in clinical settings.

**Fig. 1:**
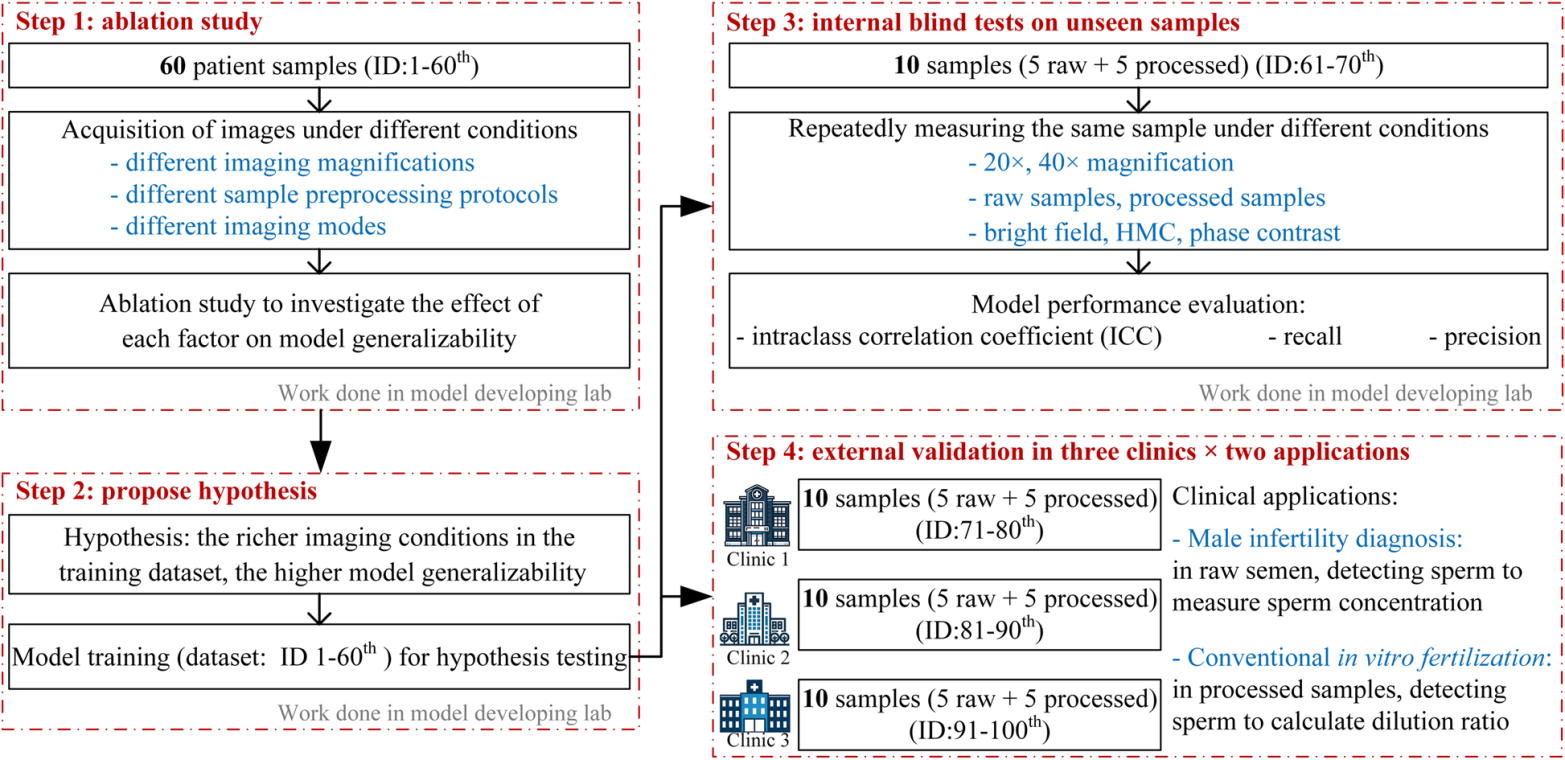
Flowchart of study design, including experiments and samples used in each section

Here we fill this knowledge gap by performing ablation studies which quantitatively revealed how model precision and recall were affected by imaging magnification, imaging mode, and sample preprocessing protocols. The workflow of this manuscript is shown in Fig. 1. As a pilot study, we evaluated performance of state-of-the-art deep learning models for detecting and identifying human sperm, due to their wide applications in andrology laboratories and IVF clinics. Based on the ablation studies, we hypothesized that improving the diversity and richness of the training dataset could increase model generalizability. This hypothesis was first tested by calculating the model’s ICC for repeated measurements on new samples. Then the hypothesis was prospectively tested via external validation in three clinics (excluding the academic lab where the model was trained) that used different image acquisition conditions and sample preprocessing protocols. The results validated the hypothesis that the richness of data in the training dataset is a key factor impacting modelgeneralizability.

**Table 1:** Summary of clinical applications of object detection models in IVF

Object	Clinical Application	Algorithm	Datasets				Reference
			Sources	Imaging mode	Resolution	Magnification	
**Sperm**	Selecting high-quality sperm during intracytoplasmic sperm injection (ICSI) treatment	YOLO	Single center	Bright field	128$\times$128	60$\times$, 40$\times$	[9]
		VGG	Multi-center	Bright field	131$\times$131	10$\times$	[10]
		VGG	Multi-center	Bright field	131$\times$131	10$\times$	[11]
		YOLO	Single center	Bright field	/	60$\times$	[46]
	Detecting sperm in semen quality analysis for male infertility diagnosis (locating sperm for subsequent measurement of sperm concentration, motility, and morphology)	YOLO	Single center	Phase contrast^a^	640$\times$480	40$\times$	[12]
		YOLO	Single center	Phase contrast	1280$\times$960	10$\times$	[13]
		YOLO	Single center	Phase contrast	640$\times$480	40$\times$	[14]
		YOLO	Single center	Phase contrast	640$\times$480	40$\times$	[43]
		YOLO	Single center	Phase contrast	640$\times$480	40$\times$	[44]
		YOLO	Single center	Hoffman modulation contrast^b^	448$\times$448	40$\times$	[45]
		YOLO	Single center	Hoffman modulation contras	1664$\times$1664	/	[35]
		YOLO	Single center	/	/	/	[36]
		YOLO	Single center	Bright field	640$\times$640	10$\times$	[37]
		YOLO VGG	Single center	Bright field	598$\times$528	20$\times$	[38]
		VGG	Single center	Bright field	150$\times$150	40$\times$	[39]
		CNN	Single center	DIC^c^	/	20$\times$,100$\times$	[47]
	Searching for sperm in testicular sperm extraction samples for azoospermia patients	YOLO	Single center	DIC	3264$\times$2448 1920$\times$1940	63$\times$	[15]
		U-Net	Single center	Bright-field Fluorescence	256$\times$256	10$\times$	[16]
**Oocyte**	Detecting oocytes for the selection of high-quality oocytes during ICSI	DeepLabV3	Single center	Bright field	1392$\times$1024	20$\times$	[17]
		U-Net	Single center	Bright field	1280$\times$1024	4$\times$,15$\times$30$\times$,40$\times$	[33]
		CNN	Single center	Bright field	250$\times$250	20$\times$	[34]
**Embryo**	Locating embryos for grading and selecting high-quality embryos for transfer	ResNet	Single center	Bright field	720$\times$480	/	[18]
		CNN	Single center	Bright field	250$\times$250	20$\times$	[20]
		YOLO	Single center	Bright field	500$\times$500	/	[40]
		VGG	Single center	Bright field	/	/	[41]
		AlexNet	Single center	Bright field	/	/	[42]
		EfficientNetV2	Single center	Bright field	1024$\times$768	/	[48]

^a^Phase contrast is a technique that enhances the contrast of transparent and colorless specimens by converting phase shifts in light passing through the specimen into changes in intensity

^b ^Hoffman modulation contrast (HMC) enhances the contrast of unstained transparent samples by modulating the phase and amplitude of transmitted light. It is commonly used for visualizing sperm and oocytes during in vitro fertilization treatment

^c ^Differential Interference Contrast (DIC) microscopy is an optical imaging technique that uses polarized light to produce high-contrast images of transparent specimens, enhancing the three-dimensional appearance of structures by exaggerating differences in optical paths

## Results

### Investigating factors that impact model generalizability

Deep learning is a data-driven approach, and the training dataset deterministically affects model performance. Considering that different clinics use different imaging conditions, we first investigated how model generalizability is affected by imaging magnification, sample preprocessing protocols, and imaging mode. Ablation study was performed where the training images for each factor was removed from the training dataset, then the model was re-trained to compare performance (Supplementary Table 1 and Supplementary Table 2). Model performance was evaluated by model precision and recall. A lower precision indicates a higher rate of false positive detection, and a lower recall indicates a higher rate of missed detection.

**Fig. 2:**
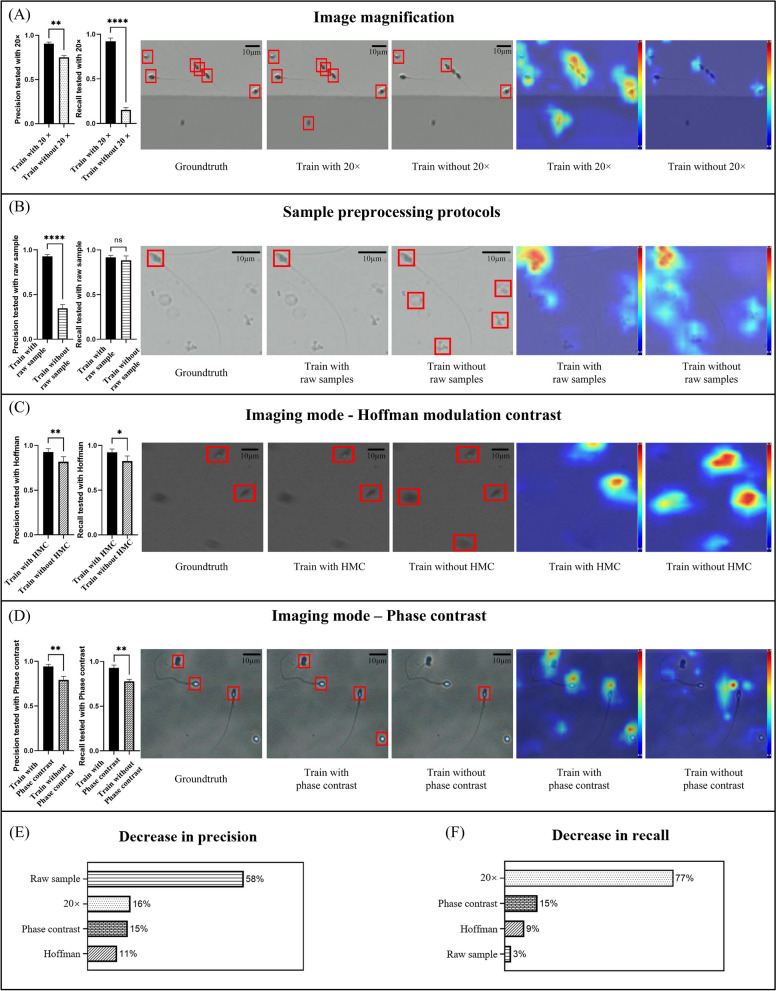
Ablation studies were performed to investigate how model generalizability is affected by imaging magnification, imaging mode, and sample preprocessing protocols. **A**-**D** In the ablation experiment, each investigated factor was removed from the training dataset and the model was re-trained to compare the precision and recall. The detection result images and visualization heatmap are also shown. Example raw sample images are shown in (**B**), and example processed sample images are shown in (**A**), (**C**), (**D**). Each scale bar represents 10$\mu m$. Each error bar represents the standard deviation of repeatedly training the model on the same dataset by three times. **E**, **F** The decrease in precision and recall caused by each factor was ranked. Removing raw sample images from the training dataset caused the largest drop in model precision, whereas removing 20$\times$ images caused the largest drop in model recall. (*$p<0.1$, **$p<0.01$, ***$p<0.001$, ****$p<0.0001$)

**Imaging magnification:** when 20$\times$ sperm images were removed from the training dataset (i.e., training the model with only 40$\times$ sperm images, but testing it with both 20$\times$ and 40$\times$ images), model precision significantly dropped from 90.64% to 75.09% ($p<0.01$, Fig. 2A). Model recall also significantly dropped from 92.08% to 15.27% ($p<0.0001$, Fig. 2A). A higher drop was observed in model recall than precision, possibly because the model learned sperm features from 40$\times$ images, and the model perceptual field cannot be mapped directly to 20$\times$ images. This interpretation was confirmed by the model weight heatmaps in Fig. 2A. The model raised less weight/attention to sperm, leading to missed detection (drop in recall).

**Sample preprocessing protocols:** when images of raw semen samples were removed from the training dataset, model precision significantly dropped by 58.11% ($p<0.0001$, Fig. 2B). Raw semen samples contained a high number of non-sperm impurities (e.g., epithelial cells, spermatocytes and leucocytes). Using only processed samples in the training dataset, the ratio between foreground (sperm) and background objects (non-sperm impurities) decreased, making the model to learn features mainly from the sperm but not enough features to distinguish the impurities. As a result, the model falsely raised more weight/attention to impurities and detected them as sperm, leading to a low precision. No significant drop in model recall was observed. This is reasonable because impurities in raw semen does not change the appearance of sperm itself, thus not causing missed detection.

**Imaging mode:** interestingly, we also noticed that when removing Hoffman modulation contrast images from the training dataset, model precision and recall also dropped (Fig. 2C). Although the drops in precision ($p<0.01$) and recall ($p<0.1$) are still significant, they are smaller than that caused by removing 20$\times$ images or raw sample images. The situation was similar for removing phase contrast images, where model precision and recall dropped by 15.01% ($p<0.01$) and 15.06% ($p<0.01$) respectively (Fig. 2D). Hoffman modulation contrast and phase contrast imaging modes mainly changed image contrast, and the resulting images were largely similar to brightfield images. Among the two experiments, the model focused on similar regions in the weight heatmaps (Fig. 2C, D).

Collectively, among all the factors, removing raw sample images caused the largest drop (58.11%, Fig. 2E) in model precision (the most false-positive detections), while removing 20$\times$ images caused the largest drop (76.81%, Fig. 2F) in model recall (the most missed detections). Removing a set of data from the training dataset reduced data richness and resulted in a decrease in both model precision and recall, confirming that richness of data in the training dataset significantly impacts model performance.

### Improving model generalizability by increasing data richness of the training dataset

Based on the ablation study, we hypothesized that increasing richness of training data would make model performance generalizable under different imaging conditions. Here data richness is twofold: 1) the training dataset should be diverse and include as many features as possible - for a model to correctly detect sperm under different imaging conditions, the model should have seen and learned such features during training to ensure a generalizable model performance; 2) the balance of foreground and background objects in the training dataset should be ensured - the lack of background objects (e.g., non-sperm impurities) decreases model precision.

To test the hypothesis, we included sperm images captured under different imaging magnifications, sample preprocessing protocols, and imaging modes into the training dataset (Supplementary Table 2). The detection model was re-trained (Fig. 4 and Supplementary Fig. 1) and its generalizability was then tested in both internal blind tests on unseen samples and external multicenter validation.

### Testing the hypothesis via internal blind test of repeated measurement on unseen samples

We first tested the hypothesis by repeatedly detecting sperm from the same sample, but under different imaging and sample preprocessing conditions. The comparison experiments were repeated on 5 raw samples and 5 processed samples. None of these samples were included in the training dataset. Generalizability was evaluated by ICC.

As summarized in Table 2, model precision and recall were both consistently around 91%, regardless of imaging magnification, imaging mode, and raw or process samples. The precision and recall values were also consistent with model training (Supplementary Fig. 1). The maximum standard deviation was 1.66% for precision and 1.77% for recall. In addition, no significant differences were observed in model precision and recall among different imaging magnifications, imaging modes or between raw samples versus process samples ($p>0.05$). Collectively, by incorporating different imaging and sample preprocessing conditions into a rich training dataset, the model achieved an ICC of 0.97 (95% CI: 0.94-0.99) for precision, and an ICC of 0.97 (95% CI: 0.93-0.99) for recall.

**Table 2:** Model performance under repeated measurements with different image acquisition conditions

Conditions		Raw sample		Processed sample	
		Precision (%)	Recall (%)	Precision (%)	Recall (%)
**Bright field**	20×	91.82±0.31	90.78±0.43	91.73±0.33	90.81±1.53
	40×	91.77±0.85	90.58±0.74	91.59±1.56	90.57±1.46
**Phase contrast**	20×	91.71±0.56	90.70±0.34	91.84±0.84	90.46±0.66
	40×	91.73±1.50	91.00±1.24	91.53±1.66	90.73±1.01
**HMC**	20×	91.91±0.52	90.60±0.25	91.53±0.98	90.54±1.77
	40×	91.63±1.62	90.50±1.47	91.76±1.21	90.44±1.02

### Testing the hypothesis via external validation among three clinics

We further performed an external multicenter clinical validation study to test model generalizability in clinical setups. The pre-trained sperm detection model was tested in three clinics, and in each clinic the model was evaluated in two clinical applications. 1) Raw semen analysis: the model was applied to detect sperm in raw semen samples. This application aids the computation of sperm concentration, which is for computer-aided sperm analysis (CASA) and the diagnosis of male infertility. 2) Processed sample analysis: the model was applied to detect sperm in processed and washed samples. This application is for calculating the dilution ratio necessary for conventional IVF treatments. In each clinic, 10 samples (including 5 raw samples and 5 processed samples) were tested, totaling 30 samples across all sites. This experimental design ensured an evaluation of the model’s performance under different sample conditions and clinical setups. The imaging setup in each clinic is summarized in Supplementary Table 3.

**Fig. 3:**
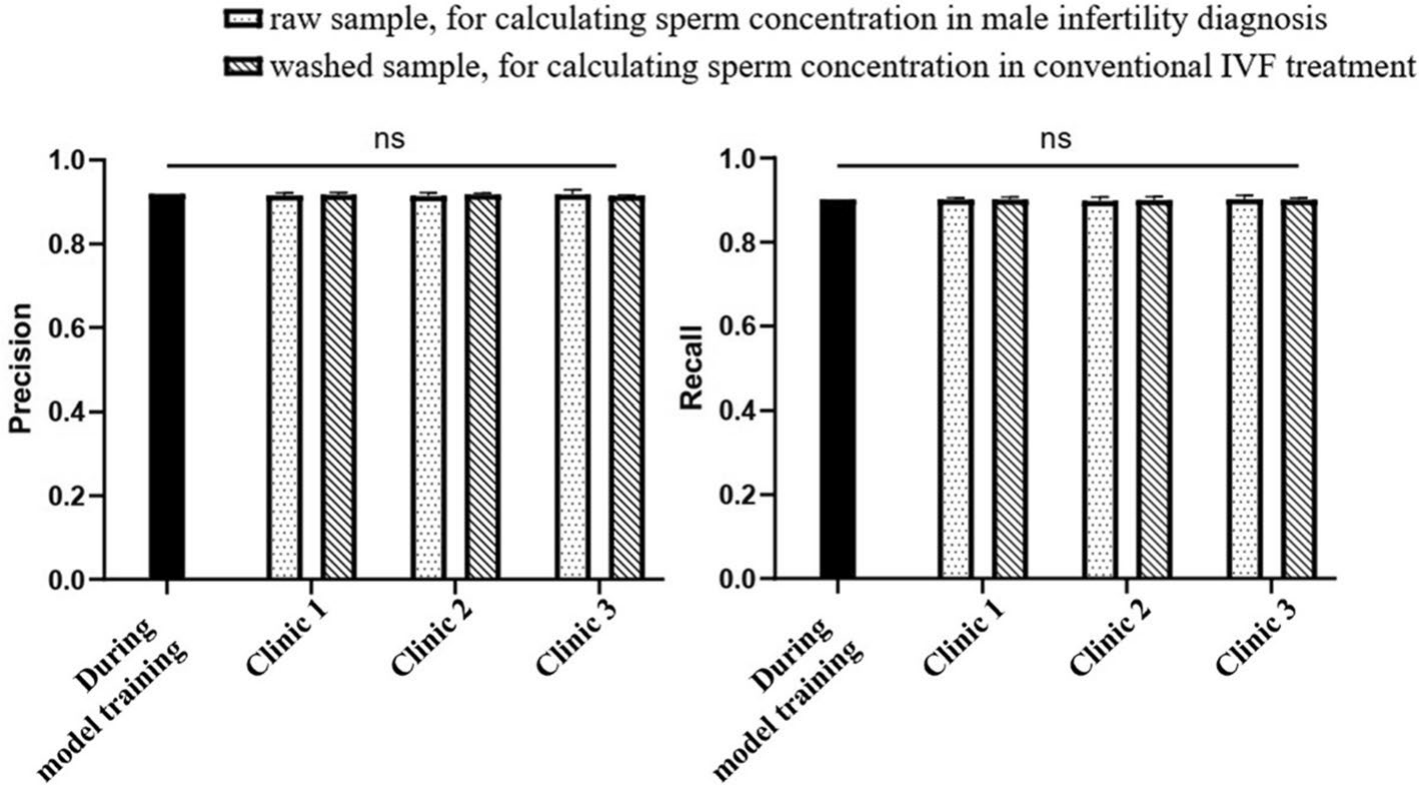
Testing the hypothesis in three clinics. The model precision and recall were tested using both raw samples and processed samples in two clinical applications. There was no significant difference in model precision and recall among three clinics as compared to the performance tested in during model training. (ns: not significant, $p>0.05$)

Detecting sperm in raw semen is challenging because of the interference of non-sperm cells in semen such as leukocytes and epithelial cells. Similar size and shape could make the algorithm incorrectly identify the sperm cells, leading to a decrease in precision, which may have an impact on sperm concentration calculation. Nonetheless, the model’s detection precision of raw samples ranged from 91.40% to 91.78% in the three clinics, and no significant differences were observed among clinics ($p>0.05$, Fig. 3). A similar result was obtained for model recall (ranged from 89.82% to 90.16%, $p>0.05$, Fig. 3).

Not surprisingly, for processed samples which had a cleaner background and less interference than raw samples, the model consistently achieved a precision ranged from 91.52% to 91.70% in the three clinics, with no significant differences among clinics ($p>0.05$, Fig. 3). Model recall for processed samples ranged from 89.98% to 90.16% ($p>0.05$). Compared with the precision and recall validated during model training, the difference in the three clinics was in the range of 0.02% to 0.20% for precision and $-0.32$% to $-0.14$% for recall, and no significant differences were observed ($p>0.05$, Fig. 3). Collectively, within each clinic, there was no significant difference between the precision or recall tested on raw samples and the processed samples ($p>0.05$, Fig. 3).

## Discussion

Sperm detection in andrology labs and IVF labs has high reproducibility requirements. Although deep learning models have been developed to automate this tedious task [54], model generalizability remains poorly understood [55]. In clinical research, this type of generalizability is also defined as conceptual reproducibility [56–59] in the literature, referring to the model’s ability to generalize and yield consistent outcomes when validating results on novel data from different sources or under various conditions. As deep learning models are increasingly applied in various clinical applications, the generalizability of such models must be investigated before they can be deployed for clinical use. Using sperm detection as a pilot study, this work 1) investigated potential factors affecting generalizability of the deep learning model, and 2) hypothesized strategies for improving the generalizability of object detection models and tested the hypothesis in multiple clinics.

For the first aim, considering deep learning is a data-driven approach and the model learns features from the provided training dataset, we investigated how the training dataset affects model generalizability. In the ablation experiments, the model was re-trained using the dataset ablating/without 20$\times$ images. When tested with 20$\times$ images as input, the re-trained model showed a significant drop in recall. The drop in recall was also observed when ablating images of raw semen and ablating images captured under the Hoffman modulation contrast and phase contrast imaging mode. These results suggest that richness of the training dataset is necessary for the model’s performance to be generalizable under different clinical setups. In other words, for the model to correctly identify an image feature during clinical deployment, the model must have seen and learned such features in the training dataset.

Interestingly, in the ablation study, we noticed that among the three factors, imaging magnification caused the largest drop in recall, with imaging mode ranked next, whereas differences in sample preprocessing protocols did not cause a significant drop (Fig. 2). One potential reason is that the appearance of sperm under 20$\times$ vs. 40$\times$ was more different than that under Hoffman modulation contrast/phase contrast vs. bright field imaging. Changing magnifications changed the number of pixels occupied by a sperm, and fewer features were available under a smaller magnification. Compared to magnification-caused changes, Hoffman modulation contrast imaging mainly changed imaging contrast and the resulting images had similar appearance to bright field images. Hence, although the targets to be detected belong to the same class of sperm, the intra-class distance [60, 61] was small for sperm images under different imaging modes and large for different magnifications. Identifying objects with a larger intra-class distance typically requires a more comprehensive and richer dataset [62, 63]. In contrast, the impurities in raw samples did not change the appearance of sperm itself, thus not causing missed detection (recall).

Another aspect of data richness is the richness of positive samples (i.e., sperm) and negative samples (i.e., background, non-sperm cells) in the training dataset. Removing the images of raw semen resulted in the largest drop in model precision. This suggests that balance of positive and negative samples should be ensured in the dataset. In the ablation experiments, the lack of negative samples such as impurities from raw semen resulted in a significantly lower precision when interferences were present. A balanced proportion of positive and negative samples can improve the anti-interference ability of the model, reduce false identification, and improve model generalizability under interference [64, 65].

**Fig. 4:**
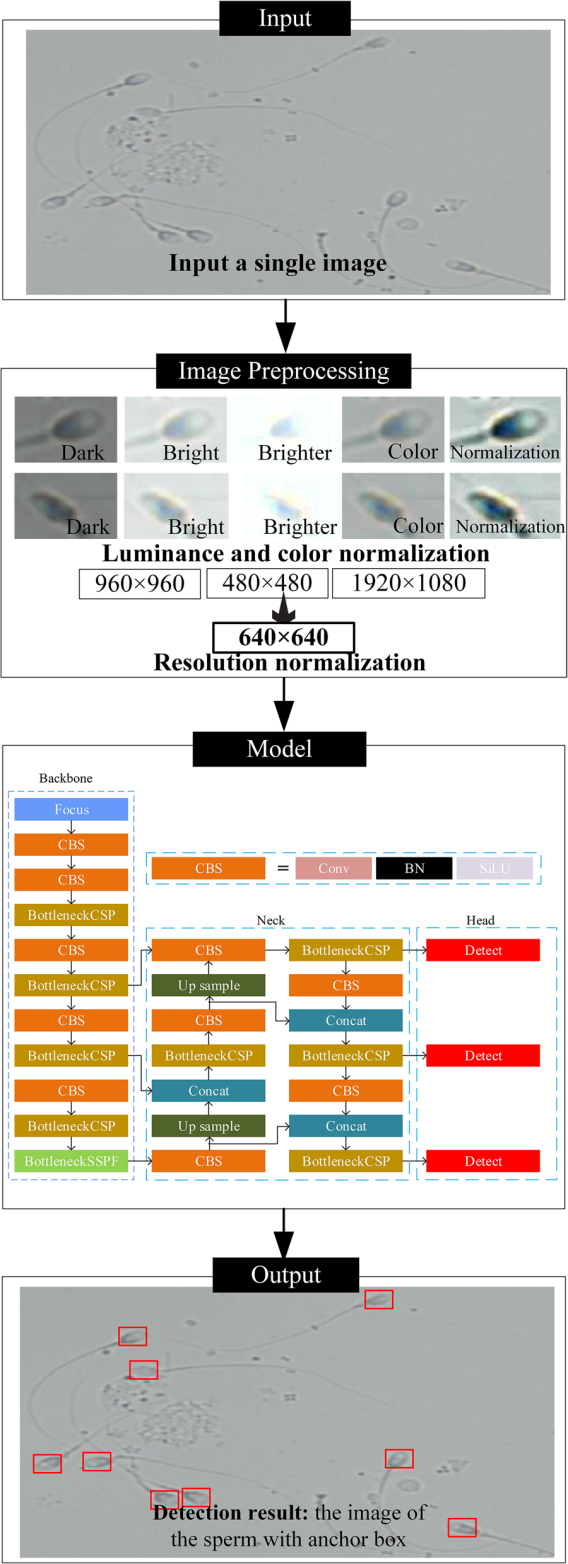
Architecture of the deep learning-based sperm detection model. The model takes a single microscopic image as input (raw sample in this example), then uses image preprocessing to normalize image luminance, resolution, and color. Sperm is detected using Yolo v5, one of the state-of-the-art convolutional neural networks for object detection. The model outputs the image of the detected with anchor box markers (bounding boxes) and coordinates of each sperm

In addition to the richness of data in the training dataset, the normalization steps during image preprocessing in the model may also contribute to model generalizability. In clinical practice, inconsistencies in the camera and image acquisition schemes lead to different brightness, color (white balance) and resolution of the acquired images. By performing image preprocessing, the brightness and color of the images can be normalized, and the resolution can be resized to the same for inputting into the model (Fig. 4), and the effect of inconsistencies in image acquisition hardware on model performance could be minimized.

For the second aim, according to the hypothesis, we re-trained the model with rich data and tested its generalizability among three clinics. It is worth noting that the objective of this work is not to create a novel model for sperm detection with improved accuracy; instead, we focused on testing the generalizability of state-of-the-art learning models under different clinical setups.

The major difference between this work and existing studies is that in addition to validating model on the retrospectively collected dataset, we further performed prospective experiments to quantify model ICC, and prospective testing among multiple clinics. In existing studies, as a routine for model development and validation, a retrospectively collected dataset is usually split into training, validation, and test sub-datasets. After each step/epoch of model training, the validation sub-dataset is fed into the model to evaluate its accuracy and precision. Hence, existing studies reported the accuracy or precision as the evaluation metric for the developed model. Although such datasets may involve data from multiple clinics, the validation and test sub-datasets were collected under the same conditions as the training sub-dataset. The lack of external validation did not allow the investigation of reproducibility metrics such as ICC.

In addition to the routine model development and validation on the sub-datasets, this work further measured model ICC by repeatedly testing the model on the same sperm samples but imaged under different image acquisition and sample processing conditions. The model achieved an ICC higher than 0.9. In further prospective multicenter validation, although each clinic used different setups, the model consistently achieved a precision and recall higher than 90%, under different image acquisition conditions (magnifications, imaging modes, camera resolution etc.) and different sample processing procedures (raw samples and processed samples).

Our results highlight the importance of considering the imaging conditions used during model development and training. As an explorative study, we aimed to comprehensively include imaging conditions to provide a complete picture of the model’s performance across various imaging settings. In practice, clinics are likely to maintain consistent imaging conditions for a given application to ensure standardization and comparability of results. When deploying deep learning models in a single clinic, it is crucial to ensure that the training data closely matches the intended use case. If a model is to be applied across multiple clinics or imaging setups, it is necessary to include a diverse range of imaging conditions in the training data to improve model generalizability.

The approach for testing a model’s generalizability from this study paves the foundation for generalizability evaluation of deep learning models in wider andrology and reproductive medicine applications. Our results also draw the attention to the training dataset of deep learning models and suggest that the richness of the training dataset directly impacts the quality of a model.

## Materials and methods

### Sample processing and dataset collection

All human semen samples were collected, processed and tested under the guidance of the World Health Organization protocol, with the approval of the ethics committee (CUHKSZ and three IVF clinics, with IRB numbers listed in section “Testing generalizability among clinics” below) and informed consent of all patients under test. Semen samples were liquefied at room temperature for 30-60 min. Raw samples were untreated, processed samples were purified by the swim-up method, and diluted to a density of 15-200$\times$ $10^6$ cells/ml density for analysis to facilitate normal medical tests. All experiments were completed within 3 hours after sperm collection.

For model training in the ablation study and hypothesis testing, a dataset containing images of 7,353 sperm from 60 semen samples was collected using a standard inverted microscope (Nikon ECLIPSE Ti2-E, Nikon Inc.) equipped with a camera (Basler MED ace 2.3, Basler Inc.). The 60 semen samples consist of 35 samples from volunteers and randomly selected medical examiners and 25 samples from infertile patients, all randomly selected, whose semen analysis parameters are summarized in Supplementary Table 1. Three embryologists annotated the sperm images and obtained the location information (i.e., bounding box) of the 7,353 sperm. The collected dataset contained images captured under two different magnifications (20$\times$, 40$\times$) and three imaging modes (bright field, Hoffman modulation contrast and phase contrast). More details of the dataset can be found in Supplementary Table 2.

### Deep learning model for sperm detection

The overall sperm detection model framework is based on YOLO v5, which is one of the state-of-the-art object detection deep learning models (Table 1). The detection model takes a single image as input, and the output is the image of the detected sperm with anchor box markers and coordinates. The neural network structure consists of a backbone module, neck module, and head module, and more details of the network can be found in Fig. 4. The acquired image resolution, luminance, and color may be different in each clinic; hence, an image preprocessing module was added to normalize these factors. The image was resized into 640$\times$640 resolution and fed into the detection model. Similarly, the luminance and color normalization step minimized their impact on model learning.

### Training of the deep learning model

The model was trained based on the dataset containing the 7,353 sperm as mentioned above (part of the dataset for ablation experiments, and the entire dataset for hypothesis testing). During training, in order to avoid overfitting, mosaic data augmentation was used to crop, arrange and stitch images randomly to augment the dataset. In training, the GIoU loss (generalized intersection over union) was used to evaluate the robustness and convergence of the model. The deep learning model was trained using the Pytorch framework (Python 3.9, Pytorch version 1.7.1), on GPU (model: NVIDIA GeForce RTX 3090 24G). The hyperparameters for training were set as follows: the optimizer was Adam, the epochs were 600, the learning rate was 0.001, and the batch size was 64.

### Visualization of model weights

To enhance the interpretability of the model, this study utilized the Gradient Weighted Class Activation Mapping (Grad-CAM) technique [66]. It is a visualization technique for understanding the decision-making process of a deep learning model in an image detection task. Grad-CAM can be integrated with common deep learning frameworks to generate class activation maps by taking a simple image as input, predicting the labels using the full model computation, inserting the global average pooling layer in the model, and computing the gradient of the feature map. The class activation maps generated by Grad-CAM visualize the regions of interest of the model on the input image. For all visualization, Grad-CAM was used in the last Conv layer of the detection model, because the last layer represents the most abstract and decision-relevant features learned by the network.

### Model evaluation

In the study, objective evaluation indicators such as precision, recall, were used to evaluate the performance of the trained sperm detection model. The calculation equations are as follows:

1 $$\begin{aligned} precision{} & {} =\frac{TP}{TP+FP} \nonumber \\ recall{} & {} =\frac{\textrm{TP}}{\mathrm {TP+FN}} \end{aligned}$$

where *TP* is the number of correctly identified sperm targets; *FP* is the number of falsely identify targets; and *FN* is the number of sperm targets that were missed by the model. In the blind test and multicenter validation, at least 200 sperm were detected in each patient sample and benchmarked against manual sperm detection results to calculate *TP*, *FP*, and *FN*.

### Testing generalizability among clinics

Model generalizability was tested among three clinics, including 1) The 3rd Affiliated Hospital of Shenzhen University in Shenzhen, China, with IRB approval number: 2021-LHRMYY-SZLL-012; 2) Reproductive & Genetic Hospital of Citic-Xiangya in Changsha, China, with IRB approval number: LL-SC2021-016; and 3) CReATe Fertility Centre in Toronto, Canada, with IRB approval number: UT35544. It is worth noting that the academic lab (CUHKSZ) for collecting the training dataset was not within these three clinics. Each clinic used a different setup for image acquisition, including different microscopes, cameras, imaging modes and magnifications. A complete list of the setup in each clinic is summarized in Supplementary Table 3.

In each clinic, 5 raw samples and 5 processed samples were processed by lab technicians. For each sample, technicians recorded videos and extracted images from them. Then the model detected the total number of sperm and benchmarked to manual results.

### Statistics

The results were expressed as means and standard deviation. No data points were excluded from the analysis. Statistical analysis was performed with MedCalc 18.3 software (MedCalc Software Ltd.). Differences between the means of two groups were tested with a two-tailed student’s t-test, and differences among more than two groups were tested by one-way analysis of variance (ANOVA), followed by Holm-Sidak pairwise comparison for normally distributed data or Dunn’s test for non-normally distributed data. Model generalizability in precision and recall was evaluated with ICC (intraclass correlation coefficient). For all tests, $p<0.05$ (labeled with an asterisk in the figures) was considered as a statistically significant difference.

### Supplementary Information

Supplementary Material 1.

## Data Availability

Data is provided within the manuscript.
