# Supplementary material for: Testing the generalizability and effectiveness of deep learning models among clinics: sperm detection as a pilot study
Source: Reprod Biol Endocrinol. 2024 May 22;22:59. doi: 10.1186/s12958-024-01232-8 (PMC11110326; doi:10.1186/s12958-024-01232-8)
Supplement: Supplementary file 1 — Supplementary Material 1. [file 12958_2024_1232_MOESM1_ESM.pdf]

Appendix A Supplementary information

This appendix contains:
Supplementary Table 1. Semen analysis statistics for two types of participants involved in the training dataset
Supplementary Table 2. Details of the collected sperm dataset for developing the deep learning model
Supplementary Table 3. List of clinics and their setups for image acquisition
Supplementary Figure 1. For hypothesis testing, the model was trained using the entire training dataset as described in Supplementary Table 2

Supplementary Table 1: Semen analysis statistics for two types of participants in the training dataset (mean±standard deviation, ns: not significant)

**Semen parameters**	**Volunteers and medical examiners**	**Infertile patients**	**Statistics**
**Number of participants**	35	25	
**PH**	7.57±0.19	7.54±0.30	ns ($p = 0.63$)
**PH** (ml)	3.76±1.52	2.77±1.26	$p < 0.01$
**Concentration** (million/ml)	93.81±58.16	111.89±152.70	ns ($p = 0.53$)
**Total sperm count** (million per ejaculation)	323.76±284.11	268.48±396.37	ns ($p = 0.54$)
**Progressive motility** (%)	50.70±8.45	26.61±17.52	$p < 0.001$
**Non-progressive motility** (%)	4.92±2.22	5.18±2.50	ns ($p = 0.67$)
**Immotile** (%)	44.67±7.24	67.82±19.42	$p < 0.001$
**Total motility** (%)	55.66±7.44	31.79±18.24	$p < 0.001$

Supplementary Table 2: The collected sperm dataset for developing the deep learning model

**Sample Type**	**Total number of sperm**	**Training sub-dataset**	**Validation sub-dataset**
Raw sample in 20x bright field	620	496	124
Processed sample in 20x bright field	642	514	128
Raw sample in 20x HMC	680	544	136
Processed sample in 20x HMC	543	434	109
Raw sample in 20x Phase contrast	540	432	108
Processed sample in 20x Phase contrast	532	426	106
Raw sample in 40x bright field	760	608	152
Processed sample in 40x bright field	654	523	131
Raw sample in 40x HMC	550	440	110
Processed sample in 40x HMC	634	507	127
Raw sample in 40x Phase contrast	583	466	117
Processed sample in 40x Phase contrast	615	492	123
Total	7353	5882	1471

Supplementary Table 3: List of clinics and their setups for image acquisition

Clinic/Laboratory	Microscope		Camera		
	Brand	Model	Brand	Model	Resolution
**Model Developing Laboratory** (The Chinese University of Hongkong, Shenzhen)	Nikon Instruments Inc.	Eclipse Ti2	Nikon Instruments Inc.	DS-Fi3	1440 x 1024
**Clinic 1** (The 3rd Affiliated Hospital of Shenzhen University)	Nikon Instruments Inc.	Eclipse Ti-S	Watec Co. Ltd.	WAT-221S	752 x 582
**Clinic 2** (Reproductive & Genetic Hospital of Citic-Xiangya)	Nikon Instruments Inc.	Eclipse Ti-S	Basler Inc.	aCA 1300-30gm	1296 x 966
**Clinic 3** (CREATE Fertility Centre)	Olympus (EVIDENT Co., Ltd)	IX83	RI (Cooper Surgical)	DC1	1920 x 1200

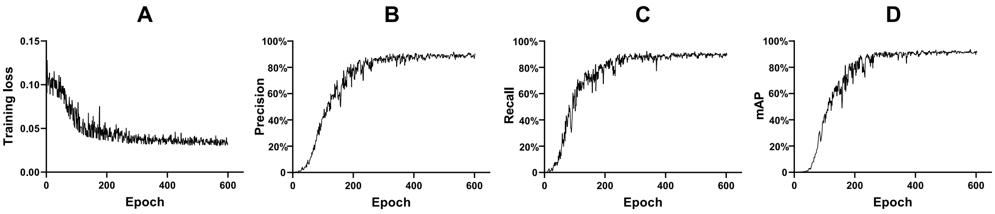

Supplementary Figure 1: **For hypothesis testing, the model was trained using the entire training dataset as described in Supplementary Table 2.** (A) The training loss decreased and converged during training. (B-D) During training, model precision ultimately reached 91.5%, model recall ultimately reached 90.3%, and model mean average precision (mAP) ultimately reached 93.5%.
